# Dopaminergic Modulation of Medial Prefrontal Cortex Deactivation in Parkinson Depression

**DOI:** 10.1155/2015/513452

**Published:** 2015-12-17

**Authors:** Anders H. Andersen, Charles D. Smith, John T. Slevin, Richard J. Kryscio, Catherine A. Martin, Frederick A. Schmitt, Lee X. Blonder

**Affiliations:** ^1^Department of Anatomy and Neurobiology, University of Kentucky, Lexington, KY 40536, USA; ^2^Magnetic Resonance Imaging and Spectroscopy Center, University of Kentucky, Lexington, KY 40536, USA; ^3^Department of Neurology, University of Kentucky, Lexington, KY 40536, USA; ^4^Sanders-Brown Center on Aging, University of Kentucky, Lexington, KY 40536, USA; ^5^Veterans Administration Medical Center, Lexington, KY 40502, USA; ^6^Department of Statistics, University of Kentucky, Lexington, KY 40536, USA; ^7^Department of Biostatistics, University of Kentucky, Lexington, KY 40536, USA; ^8^Department of Psychiatry, University of Kentucky, Lexington, KY 40536, USA; ^9^Department of Behavioral Science, University of Kentucky, Lexington, KY 40536, USA

## Abstract

Parkinson's disease (PD) is associated with emotional abnormalities. Dopaminergic medications ameliorate Parkinsonian motor symptoms, but less is known regarding the impact of dopaminergic agents on affective processing, particularly in depressed PD (dPD) patients. The aim of this study was to examine the effects of dopaminergic pharmacotherapy on brain activation to emotional stimuli in depressed versus nondepressed Parkinson disease (ndPD) patients. Participants included 18 ndPD patients (11 men, 7 women) and 10 dPD patients (7 men, 3 women). Patients viewed photographs of emotional faces during functional MRI. Scans were performed while the patient was taking anti-Parkinson medication and the day after medication had been temporarily discontinued. Results indicate that dopaminergic medications have opposite effects in the prefrontal cortex depending upon depression status. DPD patients show greater deactivation in the ventromedial prefrontal cortex (VMPFC) on dopaminergic medications than off, while ndPD patients show greater deactivation in this region off drugs. The VMPFC is in the default-mode network (DMN). DMN activity is negatively correlated with activity in brain systems used for external visual attention. Thus dopaminergic medications may promote increased attention to external visual stimuli among dPD patients but impede normal suppression of DMN activity during external stimulation among ndPD patients.

## 1. Introduction

Parkinson's disease (PD) is characterized by tremor, muscular rigidity, and bradykinesia. Individuals with PD also experience nonmotor symptoms, such as impairments in cognitive and emotional processing, including depression, anxiety, and apathy (see Blonder and Slevin [1] for a review). Although dopaminergic drugs show considerable efficacy in treating PD motor symptoms, dopaminergic pharmacotherapy may have variable effects on cognitive and affective processing depending upon the mood state of the PD patient. In particular, Blonder et al. [2] found that depressed PD (dPD) patients performed more poorly on neuropsychological tests of working memory and facial affect recognition while on dopaminergic medications than while off. Nondepressed PD (ndPD) patients showed the opposite pattern.

Functional neuroimaging studies of dPD patients have shown abnormalities in the caudate, orbitofrontal cortex, medial frontal cortex, anterior cingulate, limbic system, and thalamus [3–7]. Few studies have examined regional brain response to dopaminergic drugs during cognitive or affective processing in dPD, although functional imaging studies suggest that dopaminergic drugs modulate cognitive function among PD patients generally. (Please note that the wording “activity” is used broadly in referring to an implied underlying neural activity. Absolute levels of brain activity such as those quantified by cerebral blood flow and/or metabolism cannot be measured in fMRI experiments using BOLD contrast; only changes in activity can be detected. Positive changes in the activity level induced by a task relative to baseline are called “activation.” Negative changes in the activity level induced by a task relative to baseline are called “deactivation.” The wording “fMRI response” refers to observations of either activation or deactivation.) For example, Mattay et al. [8] found that activation in the dorsolateral prefrontal cortex, anterior cingulate, and parietal cortex during a working memory task was more focused while PD patients were on dopaminergic medications and more diffuse while they were off medication. While there were no statistically significant differences in cognitive test performance as a function of medication status, motor performance positively correlated with activation in cortical motor regions during the dopamine-replete state. Participant mood was not reported. Argyelan et al. [9], using a motor sequence learning task, showed that normal deactivation in the ventromedial prefrontal cortex (VMPFC) was suppressed in ndPD patients when they were on dopaminergic medication. Also, treatment-mediated changes in the deactivation response correlated with baseline task performance. Cools et al. [10] used PET to study PD patients on and off levodopa and found no significant differences in performance during planning and spatial working memory tasks as a function of medication status. L-Dopa decreased cerebral blood flow in the right dorsolateral prefrontal cortex during both the spatial working memory and planning tasks relative to the visuomotor control task. During the working memory task, the levodopa-induced decrease was accompanied by a significant relative increase in right occipital lobe blood flow. The authors excluded patients with a history of depression unrelated to PD, but participant mood in association with PD is not reported. Tessitore et al. [11] used fMRI to study dopaminergic modulation of affective processing in PD. They found a lack of amygdala activation in response to angry and fearful facial expressions in patients deprived of dopaminergic medications. In contrast, normal volunteers showed robust responses. Amygdala activation in PD was partially restored when dopaminergic pharmacotherapy was reinstituted. In spite of the increase in amygdala activation during the dopamine-replete state, performance on the emotional face recognition task did not differ as a function of medication status. Five of nine patients in that study had a history of depression, but the investigators did not find an association between depression and amygdala response. This may have been due to low statistical power related to small sample size.

The goal of the current study was to examine the effects of dopaminergic medication on regional brain fMRI responses to affective stimuli in depressed versus nondepressed PD patients. As described above, Blonder et al. [2] found that dPD patients showed increased depression severity based on Geriatric Depression Scale [12] scores and performed more poorly on verbal memory and facial affect recognition while on dopaminergic drugs than while off. Dopaminergic medications had the opposite effect on memory and affect recognition in ndPD patients in that performance improved on dopaminergic drugs. Past neuropsychological studies have shown impairments in affect recognition among PD patients, but these studies did not focus on comorbid depression and the results are inconsistent [13–16]. Research on depressed psychiatric patients has reported impairments in facial affect processing, suggesting that PD patients with depression may also be at increased risk for these deficits [17–21]. In healthy adults, the brain regions most typically activated on fMRI during facial affect processing include the prefrontal cortex and limbic areas implicated in dPD, providing a rationale for these tasks as salient in dPD [22].

## 2. Methods

### 2.1. Participants

The participants in this study were the same as those reported in Blonder et al. [2] and consisted of 28 right-handed, nondemented, idiopathic PD patients (10 women, 18 men). Patients were diagnosed by an American Board of Psychiatry and Neurology-certified neurologist specializing in movement disorders (JTS) using UK Parkinson's Disease Brain Bank Clinical Diagnostic Criteria [23]. Patients with a diagnosis of mild to moderate idiopathic PD (≤3 on the Hoehn and Yahr scale) were eligible to participate. Patients were under treatment with levodopa plus carbidopa either alone or in combination with dopamine agonists. Of the 28 participants, 18 were not depressed (11 men, 7 women) and 10 met DSM-IV diagnostic criteria for a depressive disorder (7 men, 3 women). Of these, seven had major depressive disorder; three had minor depression. Three of the ten dPD patients were taking selective serotonin reuptake inhibitors at the time of testing, one patient was also receiving a selective serotonin-norepinephrine reuptake inhibitor, and six were not receiving antidepressant medication. DPD patients who were taking antidepressant medication did not discontinue these medications during the study. Demographic and clinical characteristics of the participants are given in Table 1. All participants gave written informed consent under a protocol approved by the University of Kentucky Institutional Review Board. The participants in this study reflected PD patients more generally in that the dPD patients both in this sample and in the population represent slightly more than one-third of idiopathic PD patients [24] and tend to be younger [25, 26].

Participants attended a screening visit at the University of Kentucky Medical Center followed by two additional visits during which they received neuropsychological and neuroimaging assessments. During one of the two visits patients took their Parkinson medication as prescribed and during the other visit they ceased to take anti-Parkinson medication prior to midnight the night before testing. Patients on dopaminergic medications had their most recent dose approximately one to two hours prior to testing. The order of drug-on versus drug-off sessions was counterbalanced across subjects.

### 2.2. Emotional Face Processing

The functional neuroanatomy of emotional face processing has been well studied. The brain regions most strongly implicated are the fusiform gyrus, the superior temporal sulcus, the middle temporal gyrus, the medial and lateral prefrontal cortex, the anterior cingulate, and the amygdala. These are areas that have shown significant activation to neutral and emotionally expressive faces among healthy volunteers [22, 27–30]. In studies of individuals with PD, Sprengelmeyer et al. [31] found that never-medicated patients were impaired in the recognition of anger and disgust compared to medicated patients. Lawrence et al. [32] found that PD patients withdrawn from dopaminergic therapy experienced difficulty recognizing anger on the face. As described above, Tessitore et al. [11] showed a lack of amygdala activation to angry and fearful facial expressions in PD patients off dopaminergic medications. None of these studies focused on Parkinsonian depression nor did they compare depressed and nondepressed PD patients.

### 2.3. fMRI Stimuli

Stimuli consisted of grayscale still photographs of human faces from standardized sets published by Ekman and Friesen [33], Gur et al. [34, 35], and Matsumoto and Ekman [36]. Faces displayed happy, sad, angry, or neutral expressions. The choice of these expressions was based on design considerations and recent literature associating deficits in the recognition of angry faces with PD [32].

### 2.4. Presentation

Stimuli were displayed in sequence onto a translucent screen placed at the rear of the MR scanner using E-Prime software and an MRI compatible projection system. Participants viewed this screen from within the bore of the magnet by means of a mirror placed on the head coil. At the beginning of a run, pictures from each category were shown. Data acquired during this “sham” epoch were not used for analysis. Pictures were subsequently grouped three at a time into blocks according to stimulus category. Each picture was shown for 3.7 sec followed by a blank screen lasting 0.5 sec with picture epochs lasting for 12.6 sec. Picture epochs were separated by variable-duration periods of rest during which a fixation point was shown. Fixation periods lasted for 8.4–12.6 sec with a mean duration of 10.5 sec. Stimulus categories appeared in random order, and the order of presentation was counterbalanced within runs as well as across runs and sessions. Participants were told when viewing a face to concentrate on what the person is feeling and when viewing a plus sign to simply concentrate on the image and try not to think of anything else.

### 2.5. Image Acquisition and Analysis

fMRI data were collected on a Siemens Magnetom TRIO 3 Tesla imaging system using the body coil to transmit and an optional 8-channel head array coil to receive. A T_2_^*∗*^-weighted gradient echo EPI sequence was used with acquisition parameters: TR/TE = 2100/28 msec, FA = 77°, matrix = 64 × 64, and FOV = 224 × 224 mm. Each EPI volume consisted of 38 slices 3.5 mm thick, yielding isotropic voxels. Two runs were performed, each containing 185 volumes and lasting about 6.5 minutes. In the same position as the EPI images we acquired a field map using a double-echo, gradient echo technique. A 3D MPRAGE sequence (TR/TE/TI = 2100/2.93/1100 msec, FA = 12°, FOV = 224 × 256 × 192 mm, 1 × 1 × 1 mm isotropic voxels, sagittal partitions) was used to collect anatomical images for the localization of functional responses and for the registration of subjects' fMRI data sets across sessions and to stereotactic standard Talairach space [37].

Image data analysis was performed off-line using Analysis of Functional Neuroimages (AFNI) software and the Oxford Center for Functional Magnetic Resonance Imaging of the Brain (FMRIB) Software Library (FSL). The EPI volumes comprising the fMRI data were corrected for motion and slice timing within runs and registered across runs and across sessions. Geometric distortion correction of the slice images was done in native space based on the acquired field map. Voxelwise analysis of the intensity-normalized, spatially smoothed (3D Gaussian kernel), and concatenated time series response was subsequently carried out by multiple linear regression, providing simultaneous parameter estimates for each stimulus category versus a baseline of fixation comprising 80 EPI volumes. The estimated motion parameters along with drift terms were included as nuisance regressors in the baseline model. The box-car shape of the reference functions for each of the stimulus categories was convolved with Cohen's canonical hemodynamic impulse response function to better reflect the temporal delay and dynamic nature of the fMRI response. Activation contrasts of interest measured as fractional signal change were computed and transformed to standard Talairach coordinate space for second-level group analyses. Resampling by cubic spline interpolation in standard space was used, yielding isotropic 2 × 2 × 2 mm voxels. Based upon the spatial resolution of the raw fMRI data and the amount of spatial smoothing applied during preprocessing, the activation/deactivation response measured at each voxel location reflects the average across a resolution element of size 10 mm on edge. For second-level group analysis, an exploratory voxelwise random effects analysis of variance was initially carried out with depression as the between subjects factor (present or absent) and dopaminergic medication status (on or off) as the within subjects factor. The dependent response measure was the activation/deactivation contrast between emotional face stimuli (averaged across all three emotion categories of happy, angry, and sad) and a fixation baseline. Note that neutral facial expressions were not used in this analysis, as comparisons with fixation for mapping of deactivations within the default-mode network represent a more appropriate baseline in simulating resting state. The voxelwise analysis was followed by a region-of-interest (ROI) analysis using* a priori* anatomically defined ROIs and incorporating response measures for each of the separate emotions as well as controlling for potential confounds of age and education.

## 3. Results

**Figure 1:**
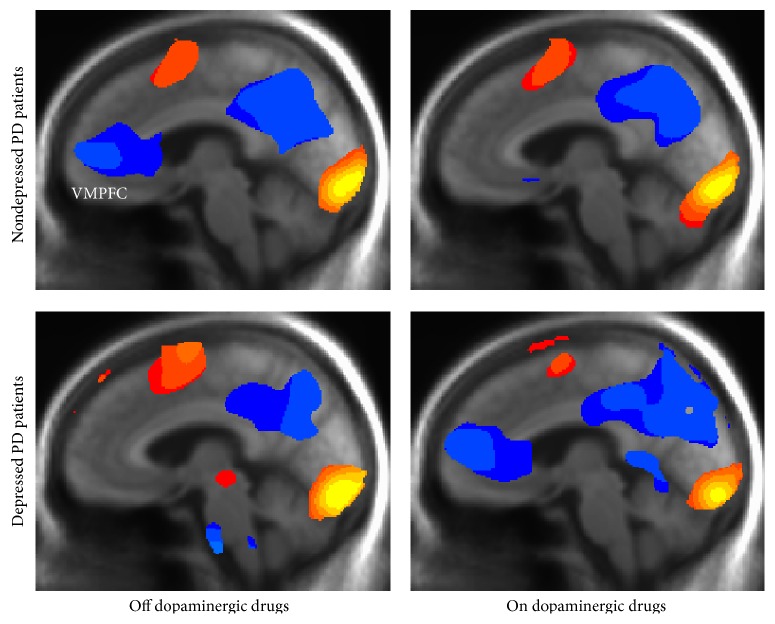
Maps thresholded at the same level of statistical significance (*P* < 0.001) showing medication-related variations in the response patterns among depressed and nondepressed PD patients.

Pronounced differences in levels of deactivation of the midline default-mode network as a function of depression and dopaminergic medication status are readily apparent (see Figure 1).

The voxelwise analysis of variance (ANOVA) revealed a significant medication by depression interaction in the ventromedial prefrontal cortex (VMPFC) with a peak effect at Talairach location [(*x*, *y*, *z*) = (3,53,16)]. DPD patients show greater deactivation on versus off dopaminergic medications while ndPD patients show the opposite pattern (see Figure 1). A companion* FreeSurfer* analysis of the structural MRI scans did not show any significant differences in medial prefrontal cortex brain region volume between the groups of nondepressed and depressed PD patients adjusted for age and education. The VMPFC location of the peak interaction effect corresponds to the anterior cingulate cortex region as defined by the automated anatomical labeling (AAL) atlas of Tzourio-Mazoyer et al. [38]. A full factorial analysis was subsequently carried out in SAS v9.3 (SAS Institute Inc., Cary, NC) using region-of-interest fMRI response data for the separate emotions extracted from this anatomical ROI, referred to in the following as VMPFC. The mean fractional signal change across all voxels within the ROI was used as the dependent measure.

**Figure 2:**
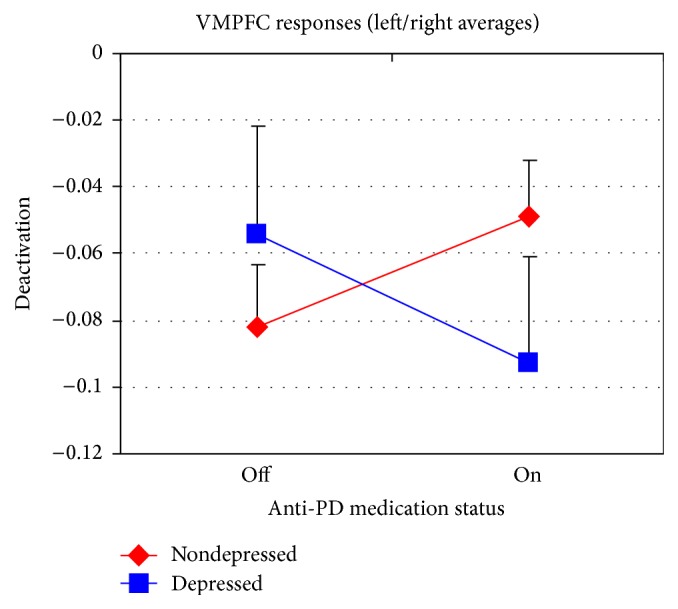
Dopaminergic medication by depression interaction in the ventromedial prefrontal cortex (VMPFC) in PD patients; data points depict the average value across emotion categories from the 4th column of Table 2. Error bars represent the standard error of the mean.

An ANOVA for a crossover design was used with depression as the between subjects factor (present or absent) and dopaminergic medication status (on or off), emotion category (angry, happy, or sad), and hemisphere (left or right) as within subjects factors. Age and education served as covariates in the analysis, although neither variable was found to be correlated with the fMRI activation/deactivation response and therefore would not affect the result. This analysis revealed a significant three-way interaction between depression, dopaminergic medication status, and emotion category (*F*(2,48) = 4.76; *P* = 0.013). The three-way interaction may be driven by a significant interaction between depression and dopaminergic medication status for happy faces (*F*(1,24) = 6.72; *P* = 0.016). There was no effect of hemisphere.  Table 2 lists the least squares marginal means averaged across hemispheres for this VMPFC region; the averages across emotion categories for the depression by medication design are displayed in Figure 2.

The separate activation/deactivation maps for patient groups and medication status clearly depict the presence of this interaction as reflecting varying levels of deactivation within the default-mode network, a network of brain regions active when the brain is at rest (see Figure 1). In particular, Figure 2 shows that depressed and nondepressed PD patients exhibit opposite effects such that dPD patients have greater deactivation in the VMPFC while on dopaminergic drugs whereas ndPD patients show greater deactivation off dopaminergic drugs. The effect of depression itself, manifested as a reduced level of VMPFC deactivation, is clearly visible in the left-hand activation/deactivation maps for facial emotion processing tasks acquired off dopaminergic medication.

**Figure 3:**
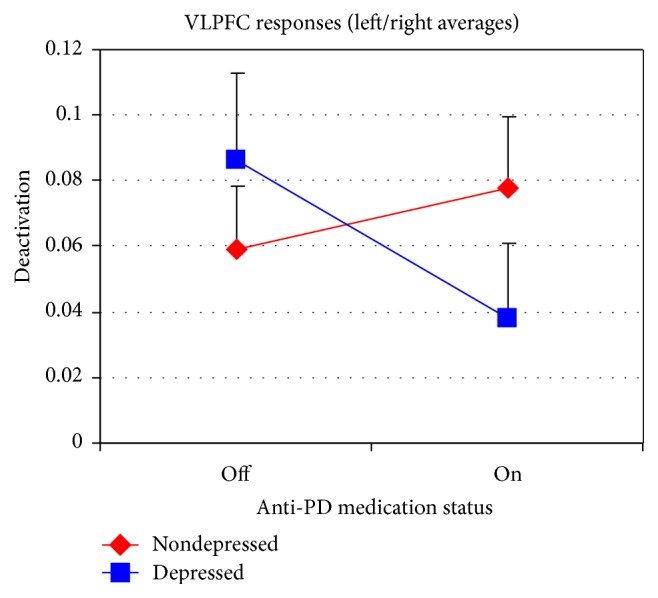
Dopaminergic medication by depression interaction in the ventrolateral prefrontal cortex (VLPFC) in PD patients. Error bars represent the standard error of the mean.

An additional interaction effect is present in the ventrolateral prefrontal cortex (VLPFC) of the voxelwise analysis bilaterally [(*x*, *y*, *z*) = (±31,27, −6)]. The effect is somewhat more pronounced and spatially extensive in the right hemisphere, although the left-right difference was not statistically significant. This location is part of the task-positive brain network activated by emotional faces and corresponds to the inferior frontal gyrus pars orbitalis region of the AAL atlas [38]. A separate ANOVA was subsequently carried out using region-of-interest fMRI response data extracted from this anatomical ROI, referred to in the following as VLPFC. The mean fractional signal change across all voxels within the ROI was again used as the dependent measure. This analysis revealed a significant depression-by-medication interaction (*F*(1,24) = 7.20; *P* = 0.013) along with a medication-by-emotion interaction (*F*(2,48) = 3.44; *P* = 0.040). Additionally, there is a significant main effect of emotion category in the VLPFC (*F*(2,48) = 4.51; *P* = 0.016) with less activation to happy faces than to angry and to sad faces.  Figure 3 shows that depressed and nondepressed PD patients exhibit opposite effects such that dPD patients have greater activation in the VLPFC while off dopaminergic drugs whereas the nondepressed PD patients show greater activation on dopaminergic drugs. The depression-by-medication interaction may be reflective of a reciprocal modulation in the prefrontal cortex between the VMPFC and VLPFC [39–42]. In both nondepressed and dPD patients, increased activation in VLPFC is associated with decreased deactivation in VMPFC.

**Figure 4:**
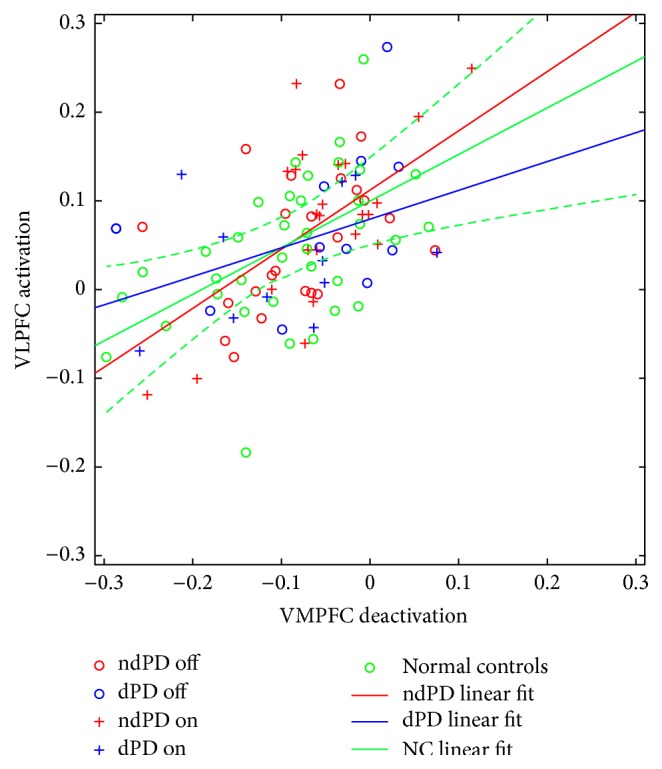
Scatter plots illustrating the relationship between VMPFC deactivation and VLPFC activation for the emotional face processing task. Observations both on and off dopaminergic medication are included. Data points for an age-matched group of normal control subjects are added for comparison. Solid lines depict fits to data from each group separately. The dashed green lines indicate the 95% confidence interval for the linear curve fitting data from a separate group of age-matched normal controls scanned under the same protocol but not part of this study.

Figure 4 depicts the bivariate nature of the (VMPFC, VLPFC) activity measures averaged across individual emotions and hemispheres for all ndPD and dPD subjects as well as off and on their dopaminergic medication. A general association between increased activation in VLPFC and decreased deactivation in VMPFC is apparent.

## 4. Discussion

In sum, we found that dopaminergic medications used to treat PD have opposite effects in two regions of the prefrontal cortex depending upon whether or not the patient suffers from depression. In particular, dPD patients show greater deactivation in the VMPFC on dopaminergic medications than they do off. In contrast, ndPD patients show greater deactivation in this region off these drugs. In the VLPFC, dPD patients show less activation on dopaminergic medications versus off, while ndPD patients show the opposite pattern.

The VMPFC is considered a region in the default-mode network (DMN), that is, a network of brain regions that remains active during rest periods [43]. Resting activity in the DMN is reduced when individuals shift attentional resources from self-referential to task-related processes. DMN activity is negatively correlated with brain systems used for focused external visual attention. Our results suggest that the administration of dopaminergic medications to dPD patients facilitates this shift in attention from self-referential to external foci whereas the administration of dopaminergic medications to nondepressed PD patients impedes this attentional shift.

Neuropsychiatric research has shown that major depressive disorder and dysthymia are associated with alterations in the structure and function of the DMN [43–49]. In PET studies of idiopathic depressed patients, the VMPFC has been shown to be hyperactive at rest [45, 46]. Similarly, an event-related fMRI design revealed an elevated tonic level of VMPFC activity [47] and abnormally increased resting-state functional connectivity in depression [48]. Hyperactivity of the VMPFC in depression is typically accompanied by reduced metabolism and blood flow within dorsal and lateral prefrontal regions such as the VLPFC [45, 50, 51]. Normal resting blood flow and metabolism in these PFC regions is restored upon treatment with antidepressants medications [52–55].

FMRI studies have found that depressed individuals show less deactivation of the VMPFC compared to normal controls during externally oriented tasks [44, 47]. For example, Sheline et al. [44] found that unmedicated patients with major depression failed to decrease activity in the DMN, including the VMPFC, when viewing and reappraising negative pictures. Johnson et al. [56] noted that the lack of deactivation in the VMPFC during distraction was positively associated with levels of negative rumination in a group of largely unmedicated depressed patients. In the present study, we see a reduced level of VMPFC deactivation in depressed compared to nondepressed PD patients off their dopaminergic medications. (Region-of-interest data for a separate group of 17 age-matched normal control subjects showed a VMPFC deactivation level of −0.105. Those subjects were scanned on two separate days without dopaminergic drugs using the same protocol but are not part of the analysis for the present study. This normal level of deactivation is greater than for either subgroup of PD patients.)

In an investigation of antidepressant effect on functional connectivity in the DMN among dysthymic patients, Posner et al. [49] found that treatment with the selective serotonin-norepinephrine reuptake inhibitor (SNRI) duloxetine normalized DMN connectivity. Posner et al. [49] noted that while DMN functional connectivity improved among dysthymic patients on SNRIs, mood did not. They attributed this apparent discrepancy to the fact that activity in the DMN is associated with rumination and the depression scale that they used did not measure that particular symptom [49, 56]. In the present study, we find greater deactivation in VMPFC and less activation in VLPFC of dPD patients when performing an emotional face recognition task on dopaminergic medications versus off, suggesting that dopaminergic drugs may act similarly to SNRIs in normalizing DMN activity patterns. Like the findings of Posner et al., medication-related changes in activity patterns did not translate into improved mood or affect recognition as these functions declined in dPD patients on dopaminergic drugs [2]; we also did not measure rumination. Hypothetically, rumination may have decreased in dPD patients on dopaminergic drugs in concert with an increase in VMPFC deactivation and presumed increased attention to affective faces. We should point out that a post hoc analysis of the subgroups of dPD patients on and off dopaminergic medication shows that the differential activation patterns seen at the group level are not due to a large confounding effect of SSRI/SNRI in half of the dPD patient group. Rather the contributions from the SSRI/SNRI medicated patients have a diluting effect on the interactions observed in the VMPFC as well as in the VLPFC.

Our findings may reflect reciprocal modulation in the prefrontal cortex between the VMPFC and VLPFC. This is consistent with prior work by Northoff et al. [41] and Harvey et al. [42]. For example, Northoff et al. [41] found that both emotional and nonemotional judgments of emotionally evocative pictures elicited increased activation in VLPFC and dorsolateral prefrontal cortex (DLPFC) and concurrent signal decreases in the VMPFC and dorsomedial prefrontal cortex. Subsequently, Northoff et al. [57] commented on the see-saw balance between medial and lateral forebrain regions. They proposed a resting-state hypothesis of major depressive disorder in which abnormal resting-state activity leads to reduced rest-stimulus interaction manifested as reduced task-related deactivation responses in those regions with high resting-state midcortical activity [41, 58]. In an fMRI study of working memory in patients with major depression, Harvey et al. [42] saw increased activation in the lateral prefrontal cortex and dorsal anterior cingulate cortex in depressed patients compared to healthy controls. Performance and reaction times were comparable. These findings suggest that depressed patients must recruit more neural resources in order to maintain working memory performance comparable to normal controls. Furthermore, Harvey et al. [42] interpreted this as an attempt by depressed subjects to counter the lack of deactivation in the limbic PFC by enhancing the activity of the lateral PFC in order to maintain the same “activity gap” between the two regions compared to healthy controls. In a review of the literature on emotion regulation, Phillips et al. [39] proposed a neural model in which VMPFC activity (among that of other medial prefrontal regions) is associated with automatic emotion regulation and VLPFC activity is associated with voluntary emotion regulation. Sheline et al. [44] noted the failure to decrease activity in the DMN in depression and suggested that dysregulation of automatic emotion processing indicates the fundamental importance of the DMN in depression. Depressed individuals achieve successful emotion regulation by recruiting additional lateral prefrontal neural regions including VLPFC to overcome the dysfunction of VMPFC [39, 40, 50]. This model is consistent with our data. In fact, it would appear that the relationship depicted for depressed and nondepressed groups of PD patients in Figures 2 and 3 holds true across a continuum; thus, our data show a linear relationship between VMPFC deactivation and VLPFC activation. The scatter plot in Figure 4 reveals that the slope of this relationship is similar for depressed and nondepressed PD patients and is similar to that for a separate group of age-matched normal controls scanned under the same protocol (but not part of this study).

We know of no prior studies that have examined the effects of dopaminergic medications on neural activity during cognitive or affective processing among dPD patients specifically. However, as noted in Introduction, a few studies have looked at cerebral activation in nondepressed PD patients or in heterogeneous samples of PD patients on and/or off dopaminergic medications [8, 10, 11], and some investigators have focused on the DMN. For example, van Eimeren et al. [59] used fMRI to examine executive function/short-term memory in the DMN of PD patients off dopaminergic medications. They found deactivation of the medial PFC in both healthy controls and PD patients. However, PD patients showed significantly less task-associated deactivation in the posterior cingulate cortex and the precuneus. In addition, the medial prefrontal cortex and the rostral ventromedial caudate nucleus were functionally disconnected in PD. The authors did not exclude patients with dysthymia or minor depression as indicated by Beck Depression Inventory cut-off scores; thus the sample was likely heterogeneous making the results difficult to interpret. Using PET, Argyelan et al. [9] observed comparable deactivation of the VMPFC during a sequence learning task in unmedicated ndPD patients and healthy control participants. When medicated, the ndPD patients failed to demonstrate learning-related deactivation based on PET. Our finding that ndPD patients show less deactivation of the VMPFC on dopaminergic medication compared to off is consistent with these results.

Delaveau et al. [60] examined PD patients' responses to emotional facial expressions on and off levodopa and found increased deactivation in the posterior cingulate/precuneus region of the DMN on medication, suggesting that levodopa increased patients' ability to attend to external stimuli. They did not see any differences in VMPFC deactivation in PD patients on and off levodopa however. While Delaveau et al. [60] excluded PD patients with major depression, they did not exclude patients with mild depression or dysthymia, providing a possible explanation for the discrepant findings.

Past research suggests that dopaminergic medication improves or impairs cognitive performance depending on the nature of the task and on individual variation in the basal level of dopamine in the underlying corticostriatal circuitry [10, 61]. Studies indicate that baseline levels of dopamine influence performance such that low levels accompany poor performance, which is generally improved by dopamine analogues or receptor agonists. By contrast, high levels of baseline dopamine accompany good performance which is generally impaired by DA receptor analogues and agonists due to a “levodopa overdose” effect [62, 63]. In our prior work, ndPD patients did more poorly on cognitive tests off dopaminergic medication, whereas dPD patients performed better off drugs [2]. On dopaminergic medication, the pattern of test scores was reversed with a resulting poorer performance among dPD subjects [2]. In the present fMRI study, activation of the VLPFC, a region known to be involved also in cognitive control, exhibits a similar interaction pattern in its modulation by depression and medication status.

Evidence suggests that genetic polymorphisms in the catechol O-methyltransferase (COMT) gene influence these responses. COMT is an enzyme that regulates dopamine and other catecholamines in various brain regions. The* met*-allele of COMT is associated with higher baseline levels of dopamine in the prefrontal cortex as well as enhanced working memory, executive function, attention, and reactivity to negative emotional stimuli on fMRI [64, 65]. The* met*-allele is also associated with a higher risk of depression [66, 67] and may indicate an increased risk of PD as well as variability in individual response to levodopa therapy [68–71]. Individuals who are homozygous for the* met*-allele (high tonic, low phasic dopamine) have been shown to perform significantly better on certain cognitive tasks than individuals possessing the* val*-allele [72].

Using fMRI with an emotion processing paradigm, Smolka et al. [64] found that the* met*-allele was associated with increased activation in VLPFC during passive viewing of unpleasant stimuli. Upon oral administration of amphetamine, which is thought to block the reuptake of dopamine, Mattay et al. [72] observed improvement in performance on an n-back working memory task among subjects with the* val* genotype while performance deteriorated in* met* subjects who have inherently high basal PFC dopamine levels. The changes seen in cognitive testing following amphetamine were accompanied by a similar switch in DLPFC activation when the n-back task was performed during fMRI data acquisition. These observations provide evidence of an inverted-U functional-response curve to increasing dopamine signaling in the PFC. Interindividual variation in the effects of dopaminergic drugs may reflect genetic variations in baseline levels of dopamine and in the individual's positioning on this inverted-U shaped curve. Individuals may therefore exhibit differential sensitivity to the positive and negative effects of dopaminergic drugs [63]. Argyelan et al. [9] in turn looked at the DMN in PD and found a reduction in VMPFC deactivation during a sequence learning task upon administration of levodopa. We see a similar reduction in the level of VMPFC deactivation in ndPD patients when performing an emotional face recognition task on dopaminergic medication compared to off. Depressed PD patients, on the other hand, increase their level of VMPFC deactivation while on dopamine analogues and agonists. When Argyelan et al. [9] performed COMT genotyping of their sample of PD patients, they noticed that the inverted-U dependence on dopamine level might explain the changes seen in VMPFC deactivation. In particular, they observed an interaction between COMT genotype and levodopa administration status in which levodopa reduced the magnitude of deactivation in* val* carriers but enhanced the deactivation response in* met* homozygotes. Given that dopaminergic input to the VMPFC from ventral striatum is relatively preserved in PD, Argyelan et al. [9] speculated that this region may be more susceptible to local overdose effects [62].

**Figure 5:**
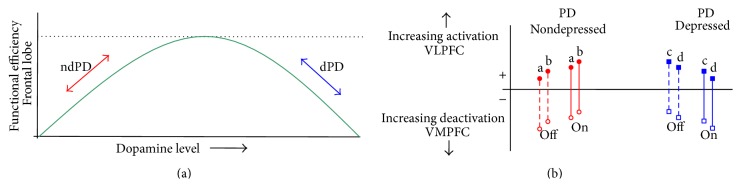
(a) Illustrating a hypothetical inverted-U dependence of frontal lobe function on dopaminergic medication associated with individual variation in baseline dopamine level. (b) Nondepressed PD patients (red) are positioned at the upstroke lower end of the curve: they reduce their level of VMPFC deactivation and increase VLPFC activation with levodopa medication. Depressed PD patients (blue) are positioned at the downstroke upper end of the curve: they increase their level of VMPFC deactivation and decrease VLPFC activation with levodopa medication. Labels a, b, c, and d indicate representative ndPD and dPD patients;* open* symbols denote the VMPFC and* closed* symbols the VLPFC brain regions; dashed lines represent the activity gap* off* dopaminergic medication, while solid lines represent that* on* levodopa.

In the present study, dPD patients off dopaminergic drugs exhibited a failure to suppress the default-mode activity manifested as a reduced level of deactivation in VMPFC during external stimulation with photographs of emotional faces. Suppression of the default-mode activity during task performance was restored by dopaminergic medication. The inverse effect involving activation of the VLPFC supports the view of reciprocal limbic-cortical function and negative mood state [51]. We posit that brain activity in the prefrontal cortex may follow an inverted-U shape with the effects of dopaminergic medication dependent upon individual variation in COMT polymorphisms that influence baseline dopamine levels (see Figure 5). If true, and in keeping with the findings of Argyelan et al. [9], we would expect an association between the* met*-allele genotype and Parkinson's depression to explain increased suppression of the VMPFC and reduced activation of the VLPFC following administration of dopaminergic medications. Future research will assess this hypothesis.

## Figures and Tables

**Table 1:** Demographic and clinical data (on PD medication).

	Nondepressed Parkinson's patients [*n* = 18; $\bar{X}$ (SD)]	Depressed Parkinson's patients [*n* = 10; $\bar{X}$ (SD)]	*P* value
Age y, mean (SD)	68.4 (8.2)	55.2 (7.0)	0.0002^∧^
Education	16.0 (3.1)	15.6 (2.3)	NS^∧^
Men/women	11/7	7/3	NS
Months since diagnosis	55.7 (39.9)	43.6 (44.0)	NS^∧^
DRS-scaled	11.0 (2.5)	12.0 (2.5)	NS^∧^
NART-R-FSIQ	107.5 (7.8)	99.1 (11.8)	0.0321^∧^
GDS-15	1.9 (1.9)	6.2 (3.4)	0.0002^∧^
Hamilton Depression Scale	4.6 (3.7)	15.0 (4.6)	<0.0001^∧^
UPDRS-Motor	16.6 (6.3)	15.0 (6.2)	NS^∧^
UPDRS-Tremor	1.1 (1.7)	0.9 (1.2)	NS^∧^
Schwab-England ADL	89.2 (11.5)	84.0 (13.5)	NS^∧^
Levodopa equivalent daily dose	513.1 (377.7)	525.0 (430.9)	NS^∧^
% on DA agonists	61.1	60.0	NS^*∗*^

Note:

DRS: Mattis Dementia Rating Scale.

NART-R-FSIQ: National Adult Reading Test-Revised, Estimated Full-Scale IQ.

GDS-15: Geriatric Depression Scale-15-Item Version.

UPDRS: Unified Parkinson's Disease Rating Scale.

^∧^ *t*-test.

^*∗*^ *χ* ^2^ test.

**Table 2:** VMPFC deactivation responses by group, medication status, and emotion category; values reflect the ROI marginal mean and standard error.

		Angry	Happy	Sad	Average
Nondepressed PD	*Off meds*	−0.104 (0.030)	−0.087 (0.030)	−0.055 (0.030)	−0.082 (0.021)
	*On meds*	−0.071 (0.030)	0.010 (0.030)	−0.085 (0.030)	−0.049 (0.021)

Depressed PD	*Off meds*	−0.044 (0.040)	−0.006 (0.040)	−0.112 (0.040)	−0.054 (0.027)
	*On meds*	−0.144 (0.040)	−0.080 (0.040)	−0.055 (0.040)	−0.093 (0.027)
